# The effect of alcohol and placebo on post-error adjustments

**DOI:** 10.3389/fnhum.2013.00003

**Published:** 2013-01-25

**Authors:** Klaas Bombeke, Nathalie Schouppe, Wout Duthoo, Wim Notebaert

**Affiliations:** Department of Experimental Psychology, Ghent UniversityGhent, Belgium

**Keywords:** alcohol, placebo, post-error slowing, post-error reduction of interference, post-error improvement of accuracy, orienting account

## Abstract

Several studies have shown detrimental effects of alcohol on post-error adjustments. In contrast to previous studies, which focused on only one aspect of post-error adaptive behavior, we compared the effect of alcohol and placebo on post-error slowing (PES), post-error reduction of interference (PERI) and post-error improvement of accuracy (PIA). Moreover, we used a between-subjects design (*N* = 45) comparing a control condition to both an alcohol and an alcohol-placebo condition as to disentangle physiological and expectancy effects of alcohol. In a standard Stroop congruency task, we found that intoxicated participants as well as participants with the incorrect belief of being intoxicated showed significant decreased PES compared to a control group. Furthermore, we found evidence for a condition-independent post-error increase of interference and post-error decrease of accuracy. The underlying mechanisms of the post-error adaptation effects are discussed in terms of the orienting account (Notebaert et al., 2009).

## Introduction

Cognitive control is the ability to detect difficulties and errors and to adjust our information processing system in order to optimize future action outcomes. A meta-analysis of Steele and Southwick (1985) suggests that alcohol typically impairs performance on tasks that involve cognitive control. More specifically, they demonstrated that alcohol has a detrimental effect in situations with simultaneous activation of conflicting responses (i.e., response conflict).

In the lab, conflict processing is typically investigated using congruency tasks, where irrelevant information may or may not interfere with response selection. In a Stroop task, for instance, participants have to respond on the basis of the color of a color-word, while the meaning of the word is irrelevant (Stroop, 1935). Participants are generally faster and more accurate on congruent (word GREEN in green ink) than on incongruent trials (word RED in green ink). Importantly, Curtin and Fairchild (2003) observed that, compared to a control group, intoxicated participants were slower and less accurate on incongruent trials, but not on congruent trials, suggesting impaired conflict processing for intoxicated participants.

Conflict processing, which takes place on incongruent trials, has been localized in posterior mediofrontal cortex (pMFC). One influential theory holds that pMFC monitors for the occurrence of conflict, after which the need for cognitive control is signaled (Botvinick et al., 2001). A role for pMFC in conflict detection has been confirmed by a large number of brain imaging studies, showing increased pMFC activity on incongruent trials (e.g., Kerns et al., 2004; Egner and Hirsch, 2005; Carter and van Veen, 2007). Interestingly, alcohol intake led to decreased pMFC activity on incongruent trials (Marinkovic and Azma, 2010; Marinkovic et al., 2011), explaining the selective deficit for incongruent trials. More important for the present study, the pMFC is also involved in the detection of errors and subsequent behavioral adjustments. Several studies have reported a correlation between pMFC activity and post-error adaptations (Gehring et al., 1993; Kerns et al., 2004; Wessel and Ullsperger, 2011). Interestingly, alcohol seems to decrease error-related activity in pMFC: the error-related negativity (ERN), an electrophysiological component related to pMFC activation (Gehring et al., 1993), was found to be reduced after alcohol consumption (Ridderinkhof et al., 2002; Bartholow et al., 2011). Taken together, these findings suggest that alcohol would also yield impaired post-error adaptation effects. Surprisingly, this has not been tested systematically.

In the error literature, three post-error adaptation effects have been reported for which different theoretical accounts have been described (for a review, see Danielmeier and Ullsperger, 2011). First, post-error slowing (PES; Rabbitt, 1966) refers to the finding that participants typically slow down after making an error. It has been suggested that PES reflects a strategy shift where participants deliberately slow down in order to decrease error likelihood in following trials (e.g., Botvinick et al., 2001). According to this account, errors will also be detected by the conflict monitoring system, in turn triggering enhanced attentional control. Alternatively, the orienting account by Notebaert et al. (2009) frames PES as an orienting reaction to infrequent errors, slowing down the following response. In line with this view, Notebaert et al. (2009) predicted that slowing would occur after an infrequent event, irrespective of the nature of that event (incorrect or correct answer). Accordingly, their results showed PES in the condition where errors were low in frequency, and post-correct slowing in the condition where errors were high frequent (see also Houtman et al., 2011). Second, it has been shown that participants' congruency effects are smaller following an error (post-error reduction of interference, PERI; Ridderinkhof et al., 2002). This effect is explained by the cognitive control account as reflecting increased attentional focusing after an erroneous response. However, the orienting account does not predict such effect, since it is presumed that attention is directed away from the task after an error. The orienting account therefore predicts an inverse PERI effect. Finally, post-error increase in accuracy (PIA) is the finding that accuracies are higher after errors than after correct trials (e.g., Laming, 1968; Marco-Pallarés et al., 2008; Danielmeier et al., 2011; Seifert et al., 2011). PIA is predicted by the cognitive control account: control is increased after an error and therefore fewer errors are committed. However, some studies have failed to find increased accuracy following errors (e.g., Hajcak et al., 2003; Hajcak and Simons, 2008; King et al., 2010) or even observed a post-error accuracy decrease (e.g., Rabbitt and Rodgers, 1977; Fiehler et al., 2005), which is more in line with the orienting account.

To date, only two studies reported the effect of alcohol on post-error behavioral adaptations, focusing only on the PERI effect. These studies showed a smaller PERI effect in an alcohol group, compared to a placebo group (Ridderinkhof et al., 2002) and a control group (Bartholow et al., 2011). No study examined alcohol effects on PES or PIA. The goal of our study was therefore to investigate the effect of alcohol on error adaptation more thoroughly. Unlike Ridderinkhof et al. (2002) and Bartholow et al. (2011), who only looked at PERI, we wanted to compare the effect of alcohol on PES, PERI, and PIA by making use of a standard congruency task. The fact that these three effects can be analyzed in one and the same task is an ideal situation to obtain knowledge about the specific ways alcohol affects cognitive processing.

We used a design that enabled us to separate the physiological and psychological effects of alcohol. The balanced placebo design (Marlatt and Rohsenow, 1980) is a 2 × 2 design consisting of two dichotomous factors: beverage manipulation and correctness of information. The beverage manipulation factor determined whether a participant had to drink an alcoholic or a non-alcoholic beverage. Correctness of information determined whether the person was correctly informed about his drinks or not (right or wrong). For ethical reasons, we did not incorporate an alcohol group that was told that they were not drinking alcohol. The alcohol-placebo condition, in which participants believed they were drinking alcohol but were in fact not, is enough to dissociate physiological from psychological effects.

## Materials and methods

### Participants

Forty-five healthy male participants (18–38 years old, *M* = 20.84, *SD* = 3.23) participated in this study. They were non-smokers and did not use medication or drugs. All participants had a normal or corrected-to-normal vision. In order to ensure the alcohol would have a similar effect on all participants, we only used “social drinkers” who drink on average between the 1.8 and 3.5 standard drinks a day (or 12.6–24.5 a week). Participants were paid for their participation and gave their permission prior to each experimental session by filling in informed consents approved by the ethical committee.

### Design

The double-blind balanced placebo design of Marlatt and Rohsenow (1980) with three groups was used. All participants were randomly distributed over the groups. The first condition (*N* = 15) was the alcohol group (*M* = 19.53 years, *SD* = 1.25 years). They had to drink alcohol and were correctly informed. The second condition (*N* = 15) was the control group (*M* = 22.8 years, *SD* = 4.52 years). They had to drink a beverage without alcohol and were also correctly informed. The third condition (*N* = 15) was the alcohol-placebo group (*M* = 20.2 years, *SD* = 2.08 years). They were informed that they were receiving alcohol but in reality the beverage contained no alcohol.

### Procedure

#### Consent and screening

Participants were asked not to use drugs or alcohol 24 h before the start of the experiment. They were also asked to abstain from drinking and eating 4 h in advance. The experiment always started at 13 or 15 h in the afternoon to avoid effects of the circadian rhythm. They were also asked to sign a document in which they gave their permission to stay at the faculty until their intoxication level was lower than the legally permitted level. In their first session, participants also had to fill in two questionnaires regarding their average alcohol consumption in daily life: the AUDIT (Saunders et al., 1993) and the Timeline follow-back questionnaire (Sobell and Sobell, 1992).

#### Beverage manipulation

The alcohol manipulation was based on Ridderinkhof et al. (2002). In the alcohol condition, participants had to drink two cups containing 0.55 g alcohol in total. Dependent on parameters like weight and alcohol tolerance, an intake of 0.55 g/kg alcohol results in a blood-alcohol concentration (BAC) of 80 mg/100 ml (= 0.7–0.8‰). By means of the formula of Widmark (Watson et al., 1981), it was determined how much vodka was needed in order to achieve a BAC of 80 mg/100 ml. This formula predicts the BAC with a number of parameters (for the exact formula, see Watson et al., 1981). We chose vodka because of its high alcohol concentration (37.5% alcohol), making it unnecessary to use more than two cups (in contradiction to beer or wine). The vodka was equally distributed in two identical cups. In each cup, green peppermint syrup (with a quantity of 75% of the amount of vodka) was added. This peppermint syrup made it impossible for the participants to taste the presence of alcohol in the cups. Furthermore, the cups were supplemented with orange juice until each cup contained 400 ml of liquid. A cover was put on each cup and participants had to drink the cocktail with a reed. In the placebo condition, the participant had to drink two 400 ml cups which contained only orange juice and mint syrup. The calculation of the amount of peppermint syrup was exactly the same as in the alcohol session. Thus, the only difference between the alcohol and placebo conditions was the presence of vodka. Participants' BAC was measured at their arrival to verify that they were completely sober and started the experiment with a BAC of zero. The drinking part of each experimental session consisted of three phases. The first 20 min, participants had to drink the first cup of 400 ml. They were asked to drink at a regular rate. The next 20 min, they had to drink the second cup of 400 ml. Finally, there was a waiting period of 20 min in order to give the alcohol the time to spread in the blood and have its physiological effect. The second BAC measurement was right before the start of the task. Furthermore, there were also measurements after every two blocks of the experimental task.

#### Task

Stimuli were presented on a 17-inch monitor with a distance of 50 cm between the screen and the participant. The experiment was programmed with T-scope software (Stevens et al., 2006). Stroop stimuli consisted of four color words (RED, GREEN, BLUE, or YELLOW), presented in red, green, blue, or yellow ink. Participants were instructed to respond to the ink color of the stimulus by pressing an associated button of a response box. Response mapping of colors was counterbalanced across participants. The instructions at the beginning of the experiment highlighted the importance of memorizing the response mapping and these instructions were repeated between every block. A trial started with the presentation of a fixation cross for 500 ms. Next, a stimulus word appeared and the participant had to react as fast and accurately as possible. The maximum response time was limited to 1500 ms. In order to avoid people forgetting the response mapping, feedback was given after every trial. A correct response was followed by the letter “J,” an incorrect response by the letter “F” and a too slow response by the letter “T.” The meaning of these letters was also explained in the instructions. Participants had to perform 10 blocks of 100 trials each. The first block was considered a practice block and would not be included in the analyses afterward. Each block was randomized and consisted of 50 congruent trials and 50 incongruent trials.

## Results

### Questionnaires

The purpose of the questionnaires was to check if the three beverage conditions did not differ from each other with regard to alcohol usage in daily life. The three conditions did not significantly differ on the AUDIT (Saunders et al., 1993), *F*_(2, 42)_ < 1, and the Timeline follow-back questionnaire (Sobell and Sobell, 1992), *F*_(2, 42)_ < 1.

### Alcohol manipulation

Because this experiment has a between-subjects design, the following analyses only concern the 15 persons in the alcohol group. At their arrival at the lab, all participants were completely sober (BAC = 0 mg/100 ml). Just before the start of the task, the average BAC was *M* = 92.6 (*SD* = 20.5) mg/100 ml. After two, four, six, eight, and ten blocks, it was respectively *M* = 98.7 (*SD* = 19.5), *M* = 101.8 (*SD* = 19.2), *M* = 101.9 (*SD* = 20.5), *M* = 100.7 (*SD* = 21.3), and *M* = 99.5 (*SD* = 20) mg/100 ml. One hour after they finished the task, the alcohol concentration of most participants fluctuated around 0.5‰.

In the post-questionnaire, all participants in the alcohol group indicated they were sure that they were drinking alcohol. In the control group, all participants were sure they were not drinking alcohol. Most importantly, all 15 participants in the alcohol-placebo group seemed to have believed they had been drinking an alcoholic beverage. This showed that the manipulation was successful and we could make valid conclusions about the different conditions.

### Post-error adjustments

The practice block and the first trial of each experimental block were removed. Next, responses that exceeded the maximum response limit (1.4%) and incorrect responses were excluded (8.4%). An outlier removal criterion of two *SD*s, calculated per participant and per congruency, was used (removal of 4.86%). The data were aggregated on the mean.

#### RT

A repeated-measures ANOVA with the within-subject variables previous accuracy (correct or incorrect) and current congruency (congruent or incongruent) and the between-subject variable beverage condition (alcohol, placebo, or control) was conducted on mean RTs. Two participants showed a low number of observations (i.e., <5) in one cell of the repeated-measures ANOVA for this analysis. However, excluding these two participants did not change the overall pattern of results. The results based on all participants are reported.

The main effect of current congruency was significant, *F*_(1, 42)_ = 101.96, *p* < 0.001, *r* = 0.84, indicating that people were faster on congruent trials (578 ms) than on incongruent trials (644 ms). The interaction between the congruency effect and condition was not significant, *F*_(2, 42)_ = 1.39, *p* > 0.1, *r* = 0.25. The main effect of condition was also not significant, *F*_(2, 42)_ < 1, *p* > 0.1, *r* = 0.18.

**Figure 1:**
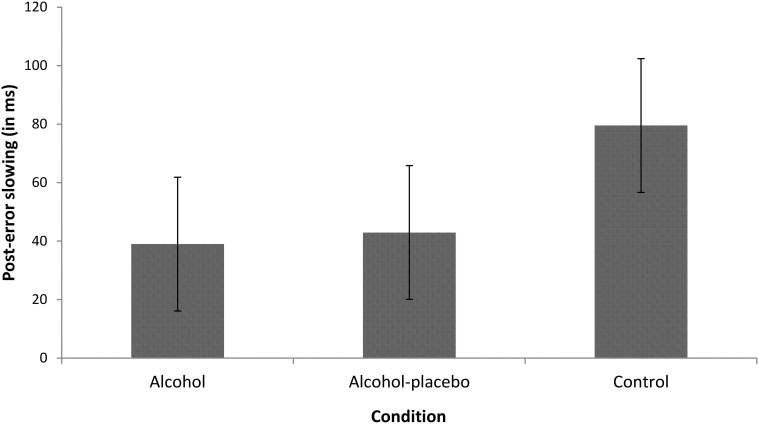
**Displays the difference in RT (in ms) between trials following incorrect and correct responses (post-error slowing) for the alcohol, alcohol-placebo, and control group.** Error bars represent 95% between-subjects confidence intervals (Loftus and Masson, 1994).

***PES.*** The main effect of previous accuracy was significant, *F*_(1, 42)_ = 68.81, *p* < 0.001, *r* = 0.79, indicating that participants were slower after an error than after a correct response (i.e., PES; see Figure 1). Furthermore, the interaction between previous accuracy and condition also turned out significant, *F*_(2, 42)_ = 3.89, *p* < 0.05, *r* = 0.39, indicating that PES differed significantly between the conditions, while PES was significant in all conditions (all *p*s < 0.001). Contrast analyses revealed that the control group showed a significantly larger PES effect (79.52 ms) than the alcohol group (38.97 ms), *F*_(1, 28)_ = 6.92, *p* < 0.05, *r* = 0.44, and a marginally significant larger PES effect than the alcohol-placebo group (42.93 ms), *F*_(1, 28)_ = 3.95, *p* = 0.057, *r* = 0.35. There was no difference between the alcohol group and the alcohol-placebo group, *F*_(1, 28)_ < 1.

**Figure 2:**
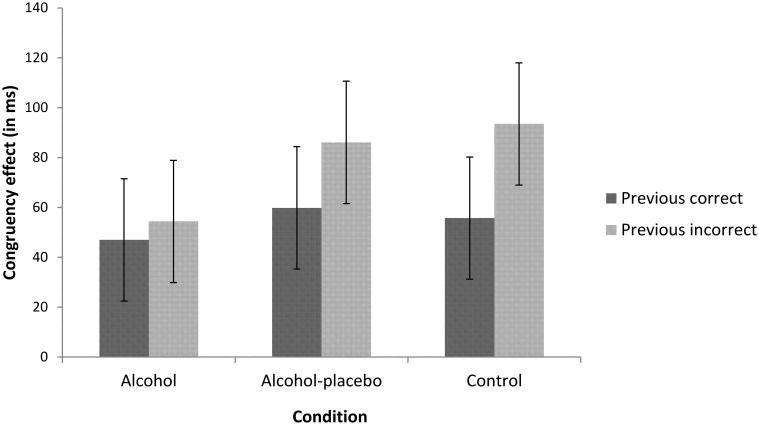
**Displays the congruency effect (incongruent–congruent) in ms for trials following correct and incorrect responses for the alcohol, alcohol-placebo, and control group.** Error bars represent 95% within-subjects confidence intervals based on the mean square error term of the interaction between previous accuracy and condition (Loftus and Masson, 1994).

***PERI.*** We found an interaction between previous accuracy and current congruency, *F*_(1, 42)_ = 5.76, *p* < 0.05, *r* = 0.35. As depicted in Figure 2, the congruency effect was larger following errors (77.97 ms) than following correct trials (54.15 ms; i.e., post-error increase of interference). This reversed PERI effect did not interact with condition, *F*_(2, 42)_ < 1, *p* > 0.1, *r* = 0.19.

#### Accuracy

A repeated measures ANOVA with the within-subject variables previous accuracy and current congruency and the between-subject variable condition was conducted on mean error rates. Overall, the error rate was on average 8.5% (*SD*=5.7%). No main effect of condition was found, *F*_(2, 42)_ < 1, *p* > 0.1, *r* = 0.15, indicating that the differences in PES across conditions were not due to different error frequencies. A main effect of current congruency was found, *F*_(1, 42)_ = 11.72, *p* < 0.001, *r* = 0.47, showing that participants were more accurate on congruent trials (7.1%) than on incongruent trials (8.4%). The interaction between current congruency and condition was not significant, *F*_(2, 42)_ < 1, *p* > 0.1, *r* = 0.13.

**Figure 3:**
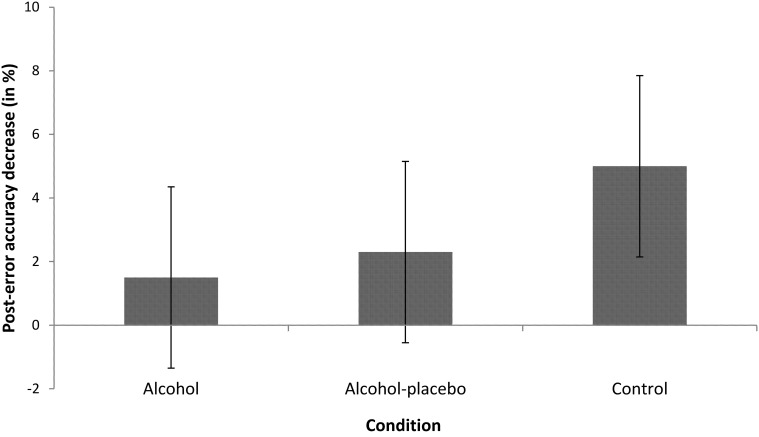
**Displays the difference in error rates (in %) between trials following incorrect and correct responses (with positive values indicating post-error accuracy decrease) for the alcohol, alcohol-placebo, and control group.** Error bars represent 95% between-subjects confidence intervals (Loftus and Masson, 1994).

***PIA.*** The main effect of previous accuracy was significant, *F*_(1, 42)_ = 11.73, *p* < 0.001, *r* = 0.47, indicating that participants were less accurate after an error (12.21%) than after a correct response (7.97%, see Figure 3). The interaction between previous accuracy and condition was not significant, *F*_(2, 42)_ = 1.20, *p* > 0.1, *r* = 0.23.

***PERI.*** The interaction between previous accuracy and congruency turned out marginally significant, *F*_(1, 42)_ = 3.16, *p* = 0.083, *r* = 0.26, indicating a larger congruency effect after an error than after a correct response. There was again no interaction between previous accuracy, congruency and condition, *F*_(2, 42)_ = 1.68, *p* ≥ 0.1, *r* = 0.27.

## Discussion

The first goal of our study was to perform a more comprehensive investigation of the effects of alcohol on post-error behavioral adaptation. Interestingly, results showed a significant condition-dependent PES effect: PES was smaller in both the alcohol and alcohol-placebo group than in the control group. Furthermore, contrary to the results of Ridderinkhof et al. (2002) and Bartholow et al. (2011) reporting a condition-dependent PERI effect, we observed a reversed PERI effect (i.e., a larger congruency effect following errors) that did not differ between groups. Similarly, we observed decreased accuracy following errors, independent of condition.

### PES

The observation that PES decreased in both the alcohol and the alcohol-placebo group supports an explanation in terms of expectancy effects: the performance of someone who beliefs to be intoxicated resembles the performance of someone who is actually intoxicated. It is important to note that the error rates in the different alcohol conditions did not differ significantly. If this would have been the case, the condition-dependent PES effect could be the result of the negative correlation between error rates and PES: the higher the error rate, the lower PES (Houtman et al., 2011). However, it appears that alcohol expectancy reduces the saliency of errors, perhaps because one expects to make more errors. This can be interpreted within the orienting account, which predicts that slowing will occur after an infrequent event, irrespectively of the nature of that event. The expectation to commit more errors intoxicated or under the impression of being intoxicated seems to have the effect that an error is no longer perceived as a surprising event.

### PERI/PIA

Whereas a cognitive control account would assume an increased focus on the task following errors, reflected in a reduced congruency effect, we found an enhanced congruency effect on post-error trials. This finding seems more consistent with the orienting account (Notebaert et al., 2009), which argues that errors or error-related processes interfere with the task at hand.

In line with this prediction, we observed reduced accuracy following errors, as was also observed in Rabbitt and Rodgers (1977) and Fiehler et al. (2005). Desmet et al. (2012), however, recently demonstrated that in a more complex, mental arithmetic task, PIA can be observed.

Although the orienting account can capture decreased accuracy and increased congruency effects after errors, it would also predict an interaction with alcohol group, in the sense that both effects should depend on error expectancy. The fact that both effects are independent of alcohol condition, suggests that increased error rates and congruency effects following errors are unrelated to the mechanism(s) responsible for PES. Such dissociation between the three post-error adaptation effects has already been reported in the literature. For instance, De Bruijn et al. (2004) demonstrated that lorezepam, a GABA_A_-inducing drug, had a selective negative effect on PERI, but not on PES. Recently, King et al. (2010) also showed that PES and PERI are mediated by different neural structures.

Taken together, our data allow for two important conclusions. First, alcohol and alcohol expectancy decrease PES. Although it is theoretically possible that this reduction in PES is caused by different factors (one physiological and one psychological, for instance), the most parsimonious explanation is that both groups show reduced PES because they both expect reduced performance, and hence increased error rates. Several recent studies have indicated that increased error rates decrease PES, presumably because errors become less salient when more errors are made (Houtman et al., 2011). Following the same logic, we argue that the expectation of making more errors also reduces the saliency of errors. Second, our data support previous studies that concluded that PES, PERI, and PIA are not caused by one and the same mechanism (e.g., King et al., 2010), as alcohol and alcohol expectancy only influences PES and not PERI or PIA.

### Conflict of interest statement

The authors declare that the research was conducted in the absence of any commercial or financial relationships that could be construed as a potential conflict of interest.
